# Using societal conditional regard to cope with drug use in the ultraorthodox community and the unintended consequences

**DOI:** 10.3389/fpsyg.2024.1344832

**Published:** 2024-04-09

**Authors:** Yael Itzhaki-Braun, Belle Gavriel-Fried

**Affiliations:** Bob Shapell School of Social Work, Tel Aviv University, Tel Aviv, Israel

**Keywords:** tight culture, substance use disorder, Ultraorthodox Jewish, societal conditional regard, self-determination theory

## Abstract

**Introduction:**

A developing theoretical framework for the investigation of tight cultures’ reaction toward members who violate communal norms is societal conditional regard (SCR).

**Methods:**

Using a qualitative interpretive approach, in the current study we investigated the way the Ultraorthodox Jewish community uses SCR to cope with substance use disorders (SUDs), which considered to be a norms violation in closed religious communities. We did so by drawing on in-depth interviews with 14 young men from the Ultraorthodox community in Israel who were diagnosed as having an SUD and were in recovery.

**Results:**

(a) The community’s socialization process, educating its members to lead a life that is the only right one; (b) The community’s use of God as the one whose love and regard are conditional; (c) The SCR emotional and behavioral practices used by the community toward individuals who violate norms, and (d) How, paradoxically, the use of SCR may eventuate in the initiation of drug use, and within the community itself.

**Discussion:**

Findings are discussed in the context of self-determination theory and SCR, and shed light on how tight cultures cope with the threat of deviation of communal norms. Implications for intervention and policy are outlined.

## Introduction

Belonging to a religious community is considered a contributing factor to an individual’s mental health, mainly due to congregational social networks (Lim and Putnam, 2010). Religious communities provide their members high levels of social capital that is expressed in solidarity, trust, support, and resources (Shapiro, 2022). According to Durkheim (1897/1951), religious communities that provide stronger forms of social control and cohesion among members help reduce negative events such as anomie and suicide. The power of believing in the same god(s) and in a shared set of religious values also helps unify members into a strongly cohesive group. In order to maintain this group, religious communities aim to ensure that people have shared expectations, abide by norms, and keep obligations, and do so by rewarding adherence to prescribed behaviors and, alternatively, by sanctioning their disregard (Shapiro, 2022). A new theoretical concept that has been developed in recent years to understand the attitude of the religious community toward members who violate community norms is societal conditional regard (SCR; Itzhaki et al., 2018; Itzhaki and Cnaan, 2021). In previous studies on norms violations in religious communities, participants who violated communal norms reported experiencing high levels of SCR from community members.

Substance use disorder (SUD) is considered to be a norms violation in religious communities in general and in the Jewish Ultraorthodox community in particular (Loewenthal, 2014). The Ultraorthodox Jewish community, is a closed religious “tight culture” (Gelfand et al., 2011), where substance use may have consequences that go beyond the physical and mental consequences for users, given the traditional social norms that derive from these communities’ religious rules (Shapiro, 2022). However, there is no scientific literature regarding the way in which the Ultraorthodox community reacts and copes with members who violate this norm and become addicted to drugs. The current study is part of a broader research project aiming to explore the paths leading to SUDs in young adults who grew up in the Ultraorthodox Jewish community (Itzhaki-Braun and Gavriel-Fried, 2022). One research question that was related to the experiences of these young adults was about community responses to their SUDs, and the answers given by the interviewees indeed correspond with the idea of SCR. Hence, using this theory as a framework, in the current study we aimed to understand how patterns of SCR are implemented in the Ultraorthodox community in order to cope with drug addiction in the community.

### Societal conditional regard

Societal conditional regard is based on the self-determination theory (SDT; Deci and Ryan, 1985; Ryan and Deci, 2000), and it focuses on community members’ attitudes as experienced by the individual. Self-determination theory (SDT) focuses on people’s inherent growth tendencies and innate psychological needs that form the basis of their self-motivation and personality integration. Ryan and Deci (2000) identified three such needs: competence, relatedness, and autonomy. By fulfilling these needs, one can experience well-being and optimal functioning. However, if these needs are not fulfilled by individuals’ immediate environment, their social and psychological development may be impaired (Moore and Hardy, 2020).

Based on STD, SCR connotes a situation in which the granting of a society’s warmth and affection is contingent upon the individual’s behaving in accordance with the society’s expectations. In societal conditional *positive* regard (SCPR), community members provide more affection, and appreciation than usual when the individual meets their expectations. In societal conditional negative regard (SCNR), community members provide less affection and appreciation than usual when the individual does not meet their expectations (Itzhaki et al., 2018). This kind of regard is a religious community’s psychological-emotional way of making its members behave in accordance with its norms, by impairing their autonomy, relatedness, and competence. Societal conditional negative regard has been found to have negative consequences, such as lower levels of well-being, whereas SCPR has been found to contribute to higher levels of well-being (Itzhaki et al., 2018; Itzhaki and Cnaan, 2021).

### Norms violations in religious communities

Religious communities expect their members to adhere to communal norms. These norms are usually based on the religious values, such as adhering to God’s commandments, not breaking up the family, and staying away from dangers such as substance use (Afifi et al., 2013; Cates and Weber, 2013; Novis-Deutsch, 2020). A violation of social norms in tight cultures can lead to being excluded from these cultures, and to the loss of social and community resources (Itzhaki et al., 2018). Specifically, in the Jewish Ultraorthodox community, community members who violate the communal norms have to cope with stigmatization, loss of communal resources, and distancing from the community (Kelly, 2014; Itzhaki et al., 2020).

Substance use disorders are considered to be a norms violation in religious communities. In recent years, the Jewish Ultraorthodox community in Israel – a closed religious community – has had to confront the issue of SUDs within its own walls. This community, representing about 12.6% of Israel’s total population (Malach and Kahner, 2020), follows the strictest interpretations of Jewish law in regard to every aspect of life (Shoham, 2012). Living in neighborhoods that are closed or separate from non-Ultraorthodox neighborhoods, the average family in these communities has six children (Israeli Central Bureau of Statistics, 2020), and are thought to comprise one of Israel’s poorest sectors (Kasir and Tsachor-Shay, 2017). The spiritual leaders are the unquestioned authority figures: They are consulted on every life issue, and their constituents almost invariably honor their decisions (Shtampper, 2017). The young people go to private, single-sex, state-certified educational institutions, and in high school (otherwise known as yeshiva), the boys focus on Jewish subjects. They continue studying at their yeshivas until they get married (Finkelman, 2011). The goal of the yeshiva high school – which plays a religious, educational, and social role – is to mold the boys’ behavior and keep them in line with the mores and values of Ultraorthodox Judaism.

Substance use disorders (SUDs), a common mental health problem among the population in general (Ouzir and Errami, 2016; Ritchie and Roser, 2022), are not limited to any specific culture or religion. Furthermore, they have wide-ranging negative consequences on several levels: for individuals, families, communities, and society as a whole (Lipari and Van Horn, 2013; World Health Organization [WHO], 2020). Although there is little knowledge in the scientific literature regarding SUDs in the Ultraorthodox community (Bar-Or et al., 2021), drug use is a direct violation of the Jewish law stipulating that life and health must be protected, and that saving a life has the highest priority. Second, drug use also violates the Ultraorthodox social norm of walking the “straight and narrow” and not deviating from the values of the Ultraorthodox community (Loewenthal, 2014). For these reasons, people who use drugs are stigmatized, and the community seeks to distance them from the community (Loewenthal, 2014; Forer et al., 2021).

## Materials and methods

This study was conducted in accordance with the qualitative interpretive approach which views human beings as agents who act with others in a sociocultural context (Morehouse, 2012), where culturally-derived interpretations supported by various theories and philosophies are used to understand the social world (Crotty, 2003). We deemed this approach suitable to studying young adults who grew up in the Ultraorthodox community and were diagnosed as having SUDs, as this religious community may represent a unique cultural context in the way it responds and relates to these young adults (as experienced by the young adults themselves).

### Sampling and recruitment

Fourteen young men were recruited by criterion sampling (Ritchie et al., 2003) from four treatment centers – under the authority of the Ministry of Welfare and Social Affairs – for addicted people. These centers are located in the center of Israel and in the Jerusalem area, and they provide services to the Ultraorthodox community. Inclusion criteria were as follows: (1) young men, over age 18, who were born and raised in an Ultraorthodox family; (2) young men diagnosed as having an SUD who received professional treatment, and (3) young men deemed “recovered,” for at least a year. The recruitment process lasted until data saturation was achieved, when the young men’s experience had been fully explored (Bowen, 2008). The participants ranged in age from 21 to 35 (*M* = 26.85). Most of them were singles, one was divorced and one was married. Ten of them live in the center of the country, and four live in the Jerusalem district. Six of the participants defined themselves as religious (light Ultraorthodox), and eight of them defined themselves as secular. However, even the secular participants claimed to believe in God. All of them had used marijuana and some had also used heroin and hashish. They had been addicted for 2 to 14 years (*M* = 5.71), and had been in recovery for 1–16 years (*M* = 4.75).

Semi-structured, in-depth interviews adhering to interview guidelines composed of open-ended questions were conducted. Participants were asked to describe their addiction process as men who grew up in the Ultraorthodox community, and the way their community treated them in light of their addiction. For example: “Please describe the reactions of your immediate environment to the addiction process” (the complete interview guide can be found in Appendix 1). The interviews lasted 45 min to 2 h; were recorded and transcribed at a later time by the authors; and were conducted between August 2020 and June 2021.

Potential interviewees were referred to the research team by the treatment centers. They were told that participation was voluntary and that non-participation would not impact their treatment (if they were in treatment) in any way. All participants were informed that the data were confidential and contingent on the participants’ written consent. In what follows, the participants’ potentially identifying information is masked, and all names have been changed to ensure anonymity. The study protocol was approved by the institutional review board of the researchers’ university.

### Data analysis

Data analysis consisted of five main phases, combining deductive and inductive content analysis. Given our research aim of understanding how SCR patterns are implemented in Ultraorthodox communities as a way to cope with SUDs, a deductive approach guided our first analysis stage (McKibben et al., 2020) in terms of selecting meaning units related to the SCR theory. Then, we used inductive coding, allowing new information to emerge from the data (McKibben et al., 2020). First, we read all interviews thoroughly, and identified all passages that presented aspects related to the different ways positive and negative SCR were implemented. Next, we conducted an inductive content analysis to enable new ideas to emerge from the data. This process included open coding and grouping the identified codes into 16 categories representing the community’s behavior toward the participants. Fourth, we aimed to construct the phenomenon, or put it back together in terms of its essential structures, by conceptualizing and writing up the findings. We probed the identified themes, using relevant literature to further our understanding. We identified four such themes as the writing-as-analysis (Richardson, 2000) process got underway. Finally, the themes were contextualized in relevant theoretical and empirical literature, in a bid to achieve both a comprehensive description of the young men’s perceptions and analytical clarity (Denzin, 2001).

### Findings

Our findings (below) are organized according to four main themes: There is only one path; The contingency of god’s love; The emotional and behavioral practices used to convey SCR; and Unintended consequences.

### There is only one path

Participants described the way the Ultraorthodox community socializes its members to adopt the values of the community. The community advocates an approach of closedness and distance from anything unrelated to the Jewish world (Goldstein and Laor, 2007). Therefore, there is no discussion of things that are prohibited, no explanations, and no giving of choices. Such indoctrination usually achieves its purpose, but those who have difficulties adhering to communal norms suffer as a result (Itzhaki et al., 2018). The participants are aware of this “only one path” phenomenon. They expressed great criticism and questioned its ability to be effective in achieving the community’s goals, as Moshe (25) said:

If you sit 300 people down and tell them, “You all love carrots,” and make them eat carrots, in the end there will be a man who tries to eat sabras [a prickly fruit found in Israel] with the thorns too. Because he doesn’t understand. They [the Ultraorthodox community] don’t let him choose. They give him only one way. This is what you love, and it’s a “must love,” because it’s faith. The mind can’t work like this. God created the mind to be capable of holding many things. He gave people the power of choice. If they do not choose, they do things without understanding why, or what is good and what isn’t good. and there it all starts…

The consequences of not adhering to the exact Ultraorthodox rules are very clear and simple, as David (26) described: “If you don’t follow the path, you’ll be left out.” This way of distancing community members who violate the communal norms is common practice among closed religious communities: It is their way of ensuring that their values and traditions are maintained for generations (Finkelman, 2011; Cates and Weber, 2013). Shmuel (28) elaborated on this way and its implications, emphasizing that his personal inability to meet the strict criteria of the Ultraorthodox community was the factor that led him to drug addiction:

The Ultraorthodox community has certain standards. There is a code for how to dress. There is the life you need to live and what is expected of you for the future, and you cannot deviate from this. There is something very exclusive in Ultraorthodox society. And if you do not fit the mold or have difficulties, you’re automatically rejected… you get lost…that’s where my addiction really started.

Yair (28) provided an image to exemplify the process that happens to youth in the Ultraorthodox community who cannot cope with this kind of indoctrination. He claimed that this suffocating way of life accomplishes the opposite of its intended goal: Instead of keeping youth away from bad influences, it makes them engage in extreme acts to break free. For him, engaging in such behavior was also his way to prove that he could:

I think it’s like a rubber band: The harder you pull, the farther it flies when you release it. The more you suffocate the boy, the farther he’ll fly when he’s released, and he’ll become more extreme. The more I was strangled, the more I needed to break free. When I moved farther away and behaved in more extreme ways, it was to say, “Look, I can.”

It seems that SCR in the Ultraorthodox community begins with early socialization processes, in which members are expected to see the Jewish religion as the community does. Participants described religious motivation in this setting as external and introjected. On the spectrum between control and autonomy, they felt they had no autonomy to choose or try something different. Their reaction to this socialization was to escape from these strict laws to other, new, unfamiliar realms, such as substance use. Their reaction is consistent with the correlation that has been found in the scientific literature between external religious motivation and substance use among youth (Hardy et al., 2020).

### The contingency of God’s love

One of the central ways participants described the community’s SCR was in their use of God; specifically, the community relays the message that God’s love and regard for His people is contingent on their behavior. This use of God may be based on the Jewish idea of “reward and punishment,” which plays a primary role in socialization processes in the Ultraorthodox community (Hakak and Rapoport, 2012; Itzhaki et al., 2018). This concept finds its origins in the Old Testament: “And if you listen to my commandments, I will give the rain of your land in its time … be careful, lest your hearts tempt you … and God will be angry with you, and stop the heavens, and there will be no rain …” (Deuteronomy, xi, xiii-xvii). That said, based on participants’ testimonials, it seems that the Ultraorthodox community has used this perception of the relationship between God and the Jewish community as a tool for controlling community members’ behavior. Shalom (34) described the way he experienced God:

I grew up in an Ultraorthodox world that was hostile, violent, that portrayed God as cruel. That if I did something wrong, I would be hurt. If I didn’t observe the Jewish commandments, I would burn in hell. I hated it all.

A very similar description could be seen in Reuven’s (27) narrative:

Through my eyes as a child, I saw God as one who punishes, who is waiting for you with a loaded gun, in case you do A, B, or C. But if you keep the commandments he’ll give you what you want. But that’s not God.

Reuven said that one of his paths to recovery was to let go of this perception of God as one whose love was contingent on people’s adherence to Jewish law:

Now [when I have recovered] I know a different God. If, before, I thought that God only loved me under certain conditions, then today I write down every morning that God loves me unconditionally. If, before, the head of the yeshiva didn’t smile or talk to me, then today God smiles at me, God loves me, God will never betray me…

In contrast to the abundant literature that emphasizes the relationship with God as a protective factor against drug use (e.g., Cheney et al., 2014; Johnson et al., 2016; Grim and Grim, 2019), the study participants felt just the opposite: They could not rely on God if His love for them was contingent on their behaving a certain way. Their addiction to drugs was a violation of the religious rules and they were therefore meant to be rejected by God. This understanding also made the community feel justified in their rejection of them.

### The emotional and behavioral practices used to convey SCR

This theme focuses on the different ways the community members made their love and affection for the participants, conditional. Throughout the interviews, a number of ways emerged. Reuven (27) shared an experience of SCNR from an admired figure. It started with this admired figure’s sudden and unexplained disregard for him. Societal conditional negative regard has many negative consequences (Itzhaki-Braun et al., 2020; Itzhaki and Cnaan, 2021), and for Reuven, the experience of SCNR actually destroyed a part of him:

I admired the head of the yeshiva. He would look at me and know how I felt. And then one day this person ignores me like I’m thin air. He was teaching, and I was sitting in front of him, and I was talking to him, and he was not responding. I used to come home and cry. My morale was shot. I told my father to see why things were suddenly like this. So he called him. and he said: “Your son belongs to an extremist sect.” What turned out? I had talked about something from Bratslav [i.e., a Hassidic stream], something I had grown up on. I was a very spiritual person. I didn’t understand the connection to God through the Gemara, but through prayer and emotion. But the head of the yeshiva didn’t like it.

Another SCNR practice was the stigmatization of those who became addicted to drugs. The addicted young men became defined solely by their addiction, as Akiva (21) described well:

In Ultraorthodox society, you’re viewed [if you’re on drugs] as ‘muktzah’ [i.e., something that is forbidden by Jewish law and from which one should keep one’s distance]. I think that something must be done so that in the end, the Ultraorthodox community will see such people not as ‘muktzah,’ but as people.

In Link and Phelan’s (2001) model of stigma, when a person is labeled as different, this label is then cognitively linked to negative stereotypes embedded in cultural beliefs. The affected individuals subsequently experience status loss and discrimination, which can result in poor outcomes (Becker et al., 2019). The Ultraorthodox community seems to think that if members know drug use will result in their being labeled “muktzah,” they will stay away from it.

Finally, we identified the SCNR practice of putting distance between problematic youth and their community. This distancing was expressed by not interacting with them, and not showing them care or concern. This reaction impaired the fulfillment of participants’ need for relatedness (Ryan and Deci, 2000). In Joshua’s (23) words:

They didn’t try to bring me back home. I had no interaction with the Ultraorthodox community throughout this whole situation. Now that I think about it, because I came from the Ultraorthodox community, I had nobody to ask, to consult with… I didn’t feel that if I reached out to someone, they would care…

For Akiva (21), the community’s distancing was not only of himself, but also of his situation: “There was no healthy interaction. Even when you tried to interact with them it wasn’t about drugs. It was only: ‘Put on a kippah’ or ‘Learn Gemara.’ Things that have nothing to do with the situation you’re in.”

Clearly, the Ultraorthodox community uses SCNR in several ways. When community members do not adhere to communal norms and expectations, they suffer the consequences in the form of hurt, stigmatization, and distancing. Based on SDT, such practices may impair the fulfillment of the three basic needs: autonomy, competence, and relatedness (Ryan and Deci, 2000).

### Unintended consequences

We called the fourth theme “unintended consequences,” as it seems that despite the widespread use of SCNR practices to keep community members away from drug use, participants’ addiction often *began* in the community. In fact, the first time they used drugs was in one of the various Ultraorthodox frameworks. When participants couldn’t manage to stay in the mainstream yeshivas, they moved to other yeshivas that were less strict. There, they met other youth who also couldn’t succeed in the mainstream yeshivas, and were already using drugs. These encounters exposed participants to drug use for the first time, as Yaacov (24) described:

I was in a yeshiva that allowed a half day of work. There were already a lot of guys who did drugs. And so I hooked up with guys who were trying to convince me, “Do drugs.” That was when all this destructive magic started.

These descriptions were repeated among many other participants as well: “*I went to a yeshiva in the north. There, I found guys who did drugs; in the middle of class they were going to do drugs, hiding*” (Shaul, 25).

David (26), however, discovered drugs not at a yeshiva itself but rather at a program for yeshiva dropouts: “*It was in the framework for dropout youth. It’s much more common there. Because it’s more open. When the outside world enters, so do drugs.*” David’s experience is consistent with findings from studies revealing the negative influence of belonging to special programs for at-risk youth in terms of increasing Ultraorthodox youths’ risk behaviors (Itzhaki-Braun and Sulimani-Aidan, 2021). These educational frameworks seem to become the basis for exposure to the outside world, especially to aspects that the Ultraorthodox community aims to keep their members away from.

## Discussion

The four themes represent the way in which SCR emerges in the Ultraorthodox community, and shed light on how tight culture cope with the threat of deviation of communal norms. The first two themes represent the communal state of mind regarding the Ultraorthodox way of life and the role of God. In line with SDT (Ryan and Deci, 2000), it seems that this state of mind suppresses the individual’s basic need for positive development. In this socialization process, which does not allow for alternative ways of thinking or behaving, the community must use strong and effective modes of social control, and it is in this fertile soil that SCR forms and begins to grow.

Seeing the Ultraorthodox way of life as the only acceptable way of life – and imposing this vision, via indoctrination, on all community members – suppresses members’ need for autonomy. Although such indoctrination may be good for most community members, for those who diverge from this way of thinking, suppressing the need for autonomy can put them at risk. For example, in a study among adolescents it was found that controlled motivation-engagement in behaviors for reasons of self-evaluative affect, or for approval from others, predicted an increase in substance use (Moore and Hardy, 2020). In our study, participants described their disagreement with this way of life and their wish to consider other ways as the factor that triggered extreme behavior such as drug use. Given that the only way of life deemed appropriate for Ultraorthodox males is studying Jewish subjects from morning to night, participants had no opportunity to choose another way. Moreover, due to the fact that the Ultraorthodox educational frameworks are not under the supervision of the state, learning disabilities tend not to be identified or treated (Barth et al., 2020). For those with learning disabilities, or those who wish to study subjects other than Jewish ones, the strict and long learning day is burdensome, and often they have no experience of success, impairing their sense of competence. Thus, in keeping with participants’ descriptions, rejecting this way of life, and succeeding in doing something not in accordance with the Ultraorthodox way of life, such as drug use, was a way to restore their sense of competence.

In addition, it seems that viewing God as conditioning His love and regard on people’s behavior impaired participants’ ability to lean on Him in difficult times, and thus hindered the fulfillment of their relatedness needs. Other studies conducted among drug users who were religious indicated that their reliance on a personal relationship with God was a major protective resource for their daily coping (Cheney et al., 2014). The participants in the current study, however, did not have this advantage. On the contrary, they perceived God’s love for them as being contingent on their behavior and therefore conceived of Him as being angry with and punishing them. It is possible that the Ultraorthodox community “uses” God in this way, to legitimize their SCNR toward members who violate the communal norms.

The third theme we identified exemplifies the specific SCR practices implemented by community members. Ignoring, labeling, and distancing – as were described by participants – are practices that impair both individuals’ need to feel competent as well as their need for relatedness to the systems that are meant to be sources of support and help. There is much literature regarding the protective role of the religious community against risky behaviors, including substance use, mainly due to the close social networks in these communities (Rasic et al., 2011; Ford and Hill, 2012; Desmond et al., 2013; Miller and Vuolo, 2018). These networks are used to help monitor risky behaviors and to exercise social supervision so as to prevent involvement in risky behaviors such as substance use (Button et al., 2010; Guo and Metcalfe, 2019). However, although the literature refers to SCPR as a social control practice which contributes to positive psychological aspects (Itzhaki et al., 2018; Itzhaki and Cnaan, 2021), the participants in the current study described only practices of SCNR. It seems that those who violate communal norms in an extreme way, receive only negative SCR, leading to the loss of social resources and impairing their relatedness needs. This attitude is in line with what tends to happen in tight cultures in the context of relationships between the individual and the community (Gelfand et al., 2011). In order to maintain the values and integrity of the religious community, the community must go against the drug users coping with deviations from the norm.

Regarding drug use, the practice of SCR has one major goal: to prevent the introduction of drugs and other risky behaviors into the Ultraorthodox community. That is, drugs and drug users have no place in the community. In line with this idea, studies have found that among religious individuals with SUDs, their substance use began at around the same time they rejected religion, the church, or God (Yeterian et al., 2018). Their transition from religiosity to secularity enhanced their experimentation with drugs and other risky behaviors (Velan and Pinchas-Mizrachi, 2019). However, we entitled our fourth theme “unintended consequences,” as the participants’ narratives made clear that, paradoxically, despite the consistent use of SCR to keep drugs out of the Ultraorthodox community, precisely the opposite eventuality ensured: The first time participants used drugs was in the Ultraorthodox community itself, obtaining them from figures in the Ultraorthodox community.

In conclusion, the study’s findings demonstrate that the Ultraorthodox community uses SCR as a way to cope with drug use, from processes of educating/socializing its members, to emotional and behavioral practices once drug use has set in. The use of SCR hinders the fulfillment of autonomy, competence, and relatedness, that are necessary for the individual’s positive development. Also, ultimately, even if SCR reduces drug exposure to some extent, it does not prevent the entry of drugs into Ultraorthodox society.

### Recommendations and limitations

The current study had a few limitations. Given the Ultraorthodox community’s insularity, and their lack of trust in “outsiders,” the recruitment process was not easy. To make it more accomplishable, the focus was on men only, as drug use in the Ultraorthodox community is more known among men than among women (Kelly, 2014). That said, future studies are encouraged to explore SCR in drug use among young women as well. Doing so would help us gain a better understanding of these processes in the general Ultraorthodox community. Furthermore, the findings of this study were analyzed through the SCR theoretical lens. Other theoretical lenses should be used in future studies.

The current study’s findings shed light on the way the Ultraorthodox Jewish community uses SCNR to control community members and keep them away from negative/risky behaviors such as drug use. It may be that, overall, SCNR is an effective practice for the Ultraorthodox community. However, for those young members who have difficulty adhering, to the communal norms, the use of SCNR is not helpful. In fact, it accomplishes precisely the opposite goal: distancing individuals from the community rather than from the drugs. Thus, we recommend using other practices to cope with drug users in the Ultraorthodox community, such as “holding” those young men who engage in risky behaviors, helping and supporting them without lending legitimacy to these behaviors. Such practices are used by community practice professionals in the treatment of Ultraorthodox divorced women and high school dropouts, the goal being to help them without undermining communal values (Itzhaki-Braun, 2021). We believe that a communal attitude of compassion and support, and providing a communal response to drug users, does not lie in opposition to the Ultraorthodox community’s rejection of risky behaviors. Finding ways to fulfill these young men’s needs for autonomy, competence, and relatedness within the community may help them recover from substance abuse without losing their communal resources. In terms of theory, we recommend that future studies investigate SCR as it relates to other addictions in the Ultraorthodox community, such as alcohol use, sex addictions, and gambling. In addition, SCR should be investigated regarding other phenomena considered norms violations, such as divorce (Barth and Ben-Ari, 2014; Band-Winterstein and Freund, 2018). Moreover, as SCR connotes a communal practice of controlling community members, it can be assumed that this practice is likely used in other tight cultures as well, such as Muslim, Amish, and Mormon. We thus recommend investigating SCR in regard to norms violations in these communities.

## Data availability statement

The datasets for this article are not publicly available due to concerns regarding participant/patient anonymity. Requests to access the datasets should be directed to the corresponding author.

## Ethics statement

The studies involving humans were approved by the Tel-Aviv University’s Review Board. The studies were conducted in accordance with the local legislation and institutional requirements. The participants provided their written informed consent to participate in this study.

## Author contributions

YI-B: Conceptualization, Data curation, Investigation, Writing – original draft, Writing – review and editing. BG-F: Conceptualization, Data curation, Investigation, Methodology, Writing – review and editing.

## Appendix 1

### Interview guide

– Please describe the reactions of your immediate environment to the addiction process.
– Has there been a specific point where someone from the community spoke to you about it? – What was the main message?
– Which relationships within the community helped you cope with addiction? Could you provide examples of this?
– Which relationships within the community made it harder for you to cope? Could you provide examples of this?
– Were there significant relationships outside the community that helped you cope with addiction?
– How did your family react to the news that you’re addicted? Could you provide examples of this?
– How did your friends react to the news that you’re addicted? Could you provide examples of this?
– How did significant figures within the community act, think, and feel toward you when they realized you’re addicted? Could you provide examples of this?
– Did you choose to share with people from the community that you’re addicted? Why specifically them? Could you provide examples of this?
– Were there figures within the community that you realized you couldn’t talk to? Why? Could you provide examples of this?

## References

[B1] Afifi T. D. Davis S. Denes A. Merrill A. (2013). Analyzing divorce from cultural and network approaches. *J. Family Stud.* 19 240–253. 10.5172/jfs.2013.19.3.240

[B2] Band-Winterstein T. Freund A. (2018). “Walking between the raindrops”: Intimate partner violence in the ultra-orthodox society in Israel from social workers’ perspective. *J. Interpers. Viol*. 33 3001–3024. 10.1177/0886260516633218 26896464

[B3] Bar-Or R. Kor A. Jaljuli I. Lev-Ran S. (2021). The epidemiology of substance use disorders among the adult Jewish population in Israel. *Eur. Addict. Res*. 27 362–370. 10.1159/000513776 33730716

[B4] Barth A. Ben-Ari A. (2014). From wallflowers to lonely trees: Divorced ultra-Orthodox women in Israel. *J. Divorce Remarriage* 55 423–440. 10.1080/10502556.2014.931756

[B5] Barth A. Ehud S. Gilad M. (2020). *One of Every Five Pupils: The Ultra-Orthodox Education System in Israel.* Jerusalem: The Israel Democracy Institute.

[B6] Becker T. Ho-Foster A. Poku O. Marobela S. Mehta H. Cao D. (2019). “It’s when the trees blossom”: Explanatory beliefs, stigma, and mental illness in the context of HIV in Botswana. *Qual. Health Res*. 29 1566–1580. 10.1177/1049732319827523 30739566 PMC7577021

[B7] Bowen G. A. (2008). Naturalistic inquiry and the saturation concept: A research note. *Qual. Res.* 8 137–152. 10.1177/1468794107085301

[B8] Button T. Hewitt J. Rhee S. Corley R. Stallings M. (2010). The moderating effect of religiosity on the genetic variance of problem alcohol use. *Alcohol Clin. Exp. Res*. 34 1619–1624. 10.1111/j.1530-0277.2010.01247.x 20569244 PMC2929317

[B9] Cates J. A. Weber C. (2013). An alcohol and drug intervention with old order Amish youth: Preliminary results of culturally segregated class participation. *J. Groups Addict. Recov.* 8 112–128. 10.1080/1556035X.2013.764199

[B10] Cheney A. Curran G. Booth B. Sullivan S. Stewart K. Borders T. (2014). The religious and spiritual dimensions of cutting down and stopping cocaine use: A qualitative exploration among African Americans in the South. *J. Drug Issues* 44 94–113. 10.1177/0022042613491108 25364038 PMC4212694

[B11] Crotty M. J. (2003). *The Foundations of Social Research. Meaning and Perspective in the Research Process.* Thousand Oaks, CA: SAGE Publications.

[B12] Deci E. L. Ryan R. M. (1985). The general causality orientations scale: Self-determination in personality. *J. Res. Pers.* 19 109–134. 10.1016/0092-6566(85)90023-6

[B13] Denzin N. K. (2001). *Interpretive Interactionism.* Thousand Oaks, CA: Sage.

[B14] Desmond S. A. Ulmer J. T. Bader C. D. (2013). Religion, self control, and substance use. *Deviant Behav.* 34 384–406. 10.1080/01639625.2012.726170

[B15] Durkheim E. (1897/1951). *Suicide: A Study in Sociology.* Los Angeles, CA: The Free Press.

[B16] Finkelman Y. (2011). “Ultra-orthodox/haredi education,” in *International Handbook of Jewish Education*, eds Miller H. Grant L. D. Pomson A. (Netherlands: Springer), 1063–1080. 10.1007/978-94-007-0354-4

[B17] Ford J. Hill T. (2012). Religiosity and adolescent substance use: Evidence from the national survey on drug use and health. *Subst. Use Misuse* 47 787–798. 10.3109/10826084.2012.667489 22443107

[B18] Forer R. Lev Bar-Or R. Sela Y. Kor A. Lev-Ran S. (2021). Stigma, treatment effectiveness, and policy: Exploring Jewish Israeli attitudes towards addiction based on a national sample. *Int. J. Ment. Health Addict.* 21 691–710. 10.1007/s11469-021-00614-y

[B19] Gelfand M. J. Raver J. L. Nishii L. Leslie L. M. Lun J. Lim B. C. (2011). Differences between tight and loose cultures: A 33-nation study. *Science* 332, 1100–1104. 21617077 10.1126/science.1197754

[B20] Goldstein S. Laor R. (2007). “Inter-cultural perspectives on the obligation to report and in identifying child abuse and neglect victims,” in *Abuse and Neglect of Children in Israel*, eds Ben-Yehuda Y. Hovav M. (Jerusalem: Ashalim), 858–888.

[B21] Grim B. Grim M. (2019). Belief, behavior, and belonging: How faith is indispensable in preventing and recovering from substance abuse. *J. Relig. Health* 58 1713–1750. 10.1007/s10943-019-00876-w 31359242 PMC6759672

[B22] Guo S. Metcalfe C. (2019). Religion as a social control: A longitudinal study of religious involvement and substance use. *Crime Delinquency* 65 1149–1181. 10.1177/0011128718787510

[B23] Hakak Y. Rapoport T. (2012). Excellence or equality in the name of God? The case of ultra-Orthodox enclave education in Israel. *J. Relig.* 92 251–276. 10.1086/663722

[B24] Hardy S. A. Nelson J. M. Frandsen S. B. Cazzell A. R. Goodman M. A. (2020). Adolescent religious motivation: A self-determination theory approach. *Int. J. Psychol. Relig.* 32 16–30. 10.1080/10508619.2020.1844968

[B25] Israeli Central Bureau of Statistics (2020). *Data of Population.* Jerusalem: Israeli Central Bureau of Statistics

[B26] Itzhaki Y. Cnaan R. A. (2021). Determinants of congregational attendees’ psychological outcomes. *J. Religion Health* 60, 1141–1159. 10.1007/s10943-019-00803-z 30919228

[B27] Itzhaki-Braun Y. (2021). The art of community social work in the ultra-orthodox community. *Br. J. Soc. Work* 51, 114–131.

[B28] Itzhaki-Braun Y. Gavriel-Fried B. (2022). “They didn’t have any idea what drugs were”: Pathways to substance use disorders among ultraorthodox Jewish males. *Int. J. Drug Policy* 109:103851. 36116337 10.1016/j.drugpo.2022.103851

[B29] Itzhaki-Braun Y. Itzhaky H. Yablon Y. B. (2020). Predictors of high-school dropout among Ultraorthodox Jewish youth. *Frontiers in Psychol*. 11:549388. 10.3389/fpsyg.2020.01911 PMC7431703 32849120

[B30] Itzhaki-Braun Y. Sulimani-Aidan Y. (2021). Risk and protective factors among at?risk ultraorthodox Jewish youth in Israel: A contextual model of positive adjustment. *Health Soc. Care Commun*. 29, 425–435. 10.1111/hsc.13102 32656880

[B31] Itzhaki Y. Itzhaky H. Yablon Y. B. (2018). The contribution of parental and societal conditional regard to adjustment of high school dropouts. *J. Adolesc*. 62, 151–161. 29197701 10.1016/j.adolescence.2017.11.014

[B32] Itzhaki Y. Yablon Y. B. Itzhaky H. (2020). Becoming less religious (BLR) and well-being among high school dropouts. *Psychol. Religion Spiritual*. 12, 45–54.

[B33] Johnson B. Lee M. Pagano M. Post S. (2016). Positive criminology and rethinking the response to adolescent addiction: Evidence on the role of social support, religiosity, and service to others. *Int. J. Criminol. Sociol*. 5 172–181. 10.6000/1929-4409.2016.05.16 28090237 PMC5234490

[B34] Kasir N. Tsachor-Shay A. (2017). *Culture and Poverty in Haredi Society.* Jerusalem: The Haredi institute for policy studies.

[B35] Kelly A. (2014). “Risk characteristics, religion and gender among Ultra-Orthodox youth – An Ecological model,” in *Children and Youth at Risk Situation in Israel*, eds Gruppper E. Romi S. (Tel Aviv: Mofet), 190–212.

[B36] Lim C. Putnam R. D. (2010). Religion, social networks, and life satisfaction. *Am. Sociol. Rev.* 75 914–933. 10.1177/0003122410386686

[B37] Link B. G. Phelan J. C. (2001). Conceptualizing stigma. *Annu. Rev. Sociol.* 27 363–385.

[B38] Lipari R. Van Horn S. (2013). *Trends in Substance Use Disorders Among Adults Aged 18 or Older.* Rockville, MD: Substance Abuse and Mental Health Services Administration 28792721

[B39] Loewenthal K. M. (2014). Addiction: Alcohol and substance abuse in Judaism. *Religions* 5 972–984. 10.3390/rel5040972

[B40] Malach G. Kahner L. (2020). *Population. The Yearbook of Ultra-Orthodox Society in Israel 2020.* Jerusalem: The Democratic Israeli Institution.

[B41] McKibben W. B. Cade R. Purgason L. L. Wahesh E. (2020). How to conduct a deductive content analysis in counseling research. *Counsel. Outcome Res. Eval.* 13 156–168. 10.1080/21501378.2020.1846992

[B42] Miller T. Vuolo M. (2018). Examining the antiascetic hypothesis through social control theory: Delinquency, religion, and reciprocation across the early life course. *Crime Delinquency* 64 1458–1488. 10.1177/0011128717750393

[B43] Moore J. Hardy S. (2020). Longitudinal relations between substance use abstinence motivations and substance use behaviors in adolescence: A self-determination theory approach. *J Pers*. 88 735–747. 10.1111/jopy.12522 31651040

[B44] Morehouse R. (2012). *Beginning interpretative inquiry: A step-by-step approach to research and evaluation*. Routledge.

[B45] Novis-Deutsch N. (2020). “God’s operating manual”: Marriage theologies in ultra-orthodox and evangelical marital guidebooks. *J. Family Hist.* 45 191–206. 10.1177/0363199019859912

[B46] Ouzir M. Errami M. (2016). Etiological theories of addiction: A comprehensive update on neurobiological, genetic and behavioural vulnerability. *Pharmacol Biochem Behav*. 148 59–68. 10.1016/j.pbb.2016.06.005 27306332

[B47] Rasic D. Kisely S. Langille D. (2011). Protective associations of importance of religion and frequency of service attendance with depression risk, suicidal behaviours and substance use in adolescents in Nova Scotia, Canada. *J. Affect. Disord*. 132 389–395. 10.1016/j.jad.2011.03.007 21458077

[B48] Richardson L. (2000). “Writing: A method of inquiry,” in *Handbook of Qualitative Research*, 2nd Edn, eds Denzin N. K. Lincoln Y. S. (Thousand Oaks, CA: Sage), 923–948.

[B49] Ritchie J. Lewis J. Elam G. (2003). Designing and selecting samples. *Qualitative Res. Methods* 77–108.

[B50] Ritchie H. Roser M. (2022). *Opioids, Cocaine, Cannabis, and Other Illicit Drugs.* Available online at: https://ourworldindata.org/drug-use (accessed March 24, 2024).

[B51] Ryan R. Deci E. (2000). Self-determination theory and the facilitation of intrinsic motivation, social development, and well-being. *Am. Psychol*. 55 68–78. 10.1037//0003-066x.55.1.68 11392867

[B52] Shapiro E. A. (2022). Protective canopy: Religious and social capital as elements of a theory of religion and health. *J. Relig. Health* 61 4466–4480. 10.1007/s10943-021-01207-8 33646492 PMC7919239

[B53] Shoham E. (2012). *Glimpse Behind the Wall: Domestic Violence in Closed Communities [Hebrew].* Beer-Sheva: Ben-Gurion University of the Negev Press.

[B54] Shtampper S. (2017). “The development of the ‘Gdolim’ phenomena,” in *The ‘Gdolim’: The Personalities Who Shaped the Face of Ultraorthodox Judaism in Israel*, eds Binyamin B. Nisim L. (Jerusalem: Magnes), 11–20

[B55] Velan B. Pinchas-Mizrachi R. (2019). Health concerns of young Israelis moving from the ultra-orthodox to the secular community: Vulnerabilities associated with transition. *Qual. Res. Med. Healthcare* 3 32–39. 10.4081/qrmh.2019.8051

[B56] World Health Organization [WHO] (2020). *WHO Reveals Leading Causes of Death and Disability Worldwide: 2000-2019.* Geneva: World Health Organization

[B57] Yeterian J. Bursik K. Kelly J. (2018). “God put weed here for us to smoke”: A mixed-methods study of religion and spirituality among adolescents with cannabis use disorders. *Subst. Abus*. 39 484–492. 10.1080/08897077.2018.1449168 29558286 PMC6430642

